# Reconstruction of novel transcription factor regulons through inference of their binding sites

**DOI:** 10.1186/s12859-015-0685-y

**Published:** 2015-09-21

**Authors:** Abdulkadir Elmas, Xiaodong Wang, Michael S. Samoilov

**Affiliations:** 1 Department of Electrical Engineering, Columbia University, 500W 120th Street, New York, 10027 NY USA; 2 Department of Bioengineering, QB3 California Institute for Quantitative Biosciences UC Berkeley, 1700 4th St #214, Berkeley, 94720 California USA

**Keywords:** Transcription factor, Regulon identification, Motif discovery, Sequential Monte Carlo filtering

## Abstract

**Background:**

In most sequenced organisms the number of known regulatory genes (e.g., transcription factors (TFs)) vastly exceeds the number of experimentally-verified regulons that could be associated with them. At present, identification of TF regulons is mostly done through comparative genomics approaches. Such methods could miss organism-specific regulatory interactions and often require expensive and time-consuming experimental techniques to generate the underlying data.

**Results:**

In this work, we present an efficient algorithm that aims to identify a given transcription factor’s regulon through inference of its unknown binding sites, based on the discovery of its binding motif. The proposed approach relies on computational methods that utilize gene expression data sets and knockout fitness data sets which are available or may be straightforwardly obtained for many organisms. We computationally constructed the profiles of putative regulons for the TFs LexA, PurR and Fur in *E. coli K12* and identified their binding motifs. Comparisons with an experimentally-verified database showed high recovery rates of the known regulon members, and indicated good predictions for the newly found genes with high biological significance. The proposed approach is also applicable to novel organisms for predicting unknown regulons of the transcriptional regulators. Results for the hypothetical protein *D* *d* *e*0289 in *D. alaskensis* include the discovery of a Fis-type TF binding motif.

**Conclusions:**

The proposed motif-based regulon inference approach can discover the organism-specific regulatory interactions on a single genome, which may be missed by current comparative genomics techniques due to their limitations.

**Electronic supplementary material:**

The online version of this article (doi:10.1186/s12859-015-0685-y) contains supplementary material, which is available to authorized users.

## Background

In most sequenced genomes a significant proportion (3–6 %) of all genes are known to encode transcription factors [1], an essential DNA-binding component that regulates target gene transcriptional activity. The promoter regions where TFs specifically bind on genome are usually located in intergenic sites. Extensive sequencing of genomes of various organisms revealed that there is a large conservation of intergenic regions across different species, often occurring among moderately-distant relatives. This is the main intuition behind comparative genomics approaches where one aims to reconstruct regulatory networks by exploiting evolutionary conservation of regulatory features. The assumption is that if a TF-encoding gene is preserved in a set of closely-related species, the respective target genes that are regulated via cognate TF binding sites also tend to be preserved [2]. Such regulatory elements as TFBSs and their target genes identified for each genome constitute the “*regulon*” of the given TF.

Although most known regulators abide evolutionary conservation, many TF-encoding genes can be organism-specific due to various reasons and the orthologs may not exist in closely-related species. In particular, the discovery of horizontal gene transfer can explain the occurrence of nonconserved regulatory members [3]. The intuition of “*true sites occur upstream of orthologous genes, false sites are scattered at random*” [2] can thereby miss organism-specific interactions by treating them as false predictions. Hence, there is some limitation to the comparative genomics approaches, and alternative techniques are needed to identify organism-specific regulatory interactions [4].

In this work, we attack this problem from a more general computational perspective by aiming at single-genome TF regulon reconstruction, which makes our approach also suitable for novel organisms. We demonstrated our results for the TFs LexA, PurR and Fur in model bacteria *Escherichia coli K12* by comparing to their respective regulons in manually curated RegPrecise database [5]. The extended predictions –which are not captured by RegPrecise– are presented with annotations provided by GO [6] and Protein Interactions (http://www.pir.uniprot.org) databases. Putative regulon genes reported with high biological significance have expanded the known regulons of LexA, PurR and Fur. Furthermore, the results for a novel genome *Desulfovibrio alaskensis* discovered a Fis-type motif for the hypothetical regulator *D* *d* *e*0289.

### Motif-based inference of novel regulons of transcription factors

The *cis*-acting regulatory elements of genes are usually located in upstream regions of their coding sequences, where gene expression is controlled by sequence-specific binding of the TFs. Co-expressed genes that have similar TF binding patterns in their regulatory regions can be good candidates for a putative regulon. Binding preference (motif) of a TF can be described by a matrix that represents the frequency of nucleotides observed in each position of the known binding sites. Among others, the position weight matrix (PWM) is a well-suited representation of motifs for statistical evaluation of the corresponding binding sites [7], and it is also a more sensitive metric for TFBS recognition [8].

Recently, more complex models are introduced when modeling TF-DNA binding affinities. In [9], it is shown that DNA structural features can be calculated from the nucleotide sequences in motif databases, and later [10] et al. proposed that certain 3D DNA shape information can be derived from high-throughput approaches. In [11], epigenetic factors (methylation, histone modification etc.) are considered in TF binding, where they investigated certain location- and cell-type specific relationships between epigenetic modifications and binding affinities. Although these studies expand the knowledge for modeling TF binding affinity, the proposed methods may not be readily employed in every genome. In this study, we focused on a more general regulon recovery approach based on the discovery of sequence motifs that could be broadly applied by only using the genome sequence and corresponding gene expression data sets.

**Fig. 1:**
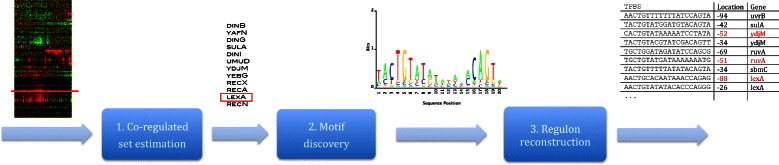
Motif-based inference of novel regulons of transcription factors

We present an integrated method for motif-based inference of novel regulons of transcription factors (Fig. 1). For a given TF (and its coding gene), the putatively co-regulated gene set is estimated by utilizing available gene expression and knockout fitness data sets in the proposed biclustering method. The approach proceeds by performing motif discovery in the upstream sequences of this high-confidence gene set. For this, we developed a probabilistic algorithm BAMBI2b see Additional file 5 that can estimate, from the supplied sequences, the main regulatory factor’s unknown weight matrix of unknown length and unknown intrinsic sequence symmetry. Once the motif is obtained the entire genome is scanned by it for TFBS prediction, where the putative TF-DNA binding affinities are estimated by statistical over-representation of the elucidated motif. Finally, the candidate genes located in the downstream of the predicted TFBSs are checked and identified as members of the putative regulon.

In *Methods* section, we provide an overview of the key steps in our approach and general descriptions of the implemented algorithms. The mathematical details are given in the Additional file 1.

By using the proposed approaches we estimated binding motifs of the given transcription factors, and reconstructed their putative binding sites and regulons. We compared our results (i.e., estimated motifs, binding sites and regulated genes) with the RegPrecise database which is manually curated by an approach [12] most relevant to our work. We used the well-studied transcription factor regulons, LexA, PurR, and Fur in the model organism *E. coli* to validate our predictions. LexA is a repressor protein that –under non-stress conditions– represses SOS response genes which involve in repairing DNA damages. Manually-curated LexA regulon in RegPrecise database consists of 30 genes that are regulated by 26 operons. PurR is an important repressor for the transcriptional regulation of purine metabolism. Its regulon includes genes participating in the biosynthesis of purine/pyrimidine nucleotides. FUR consists of a family of TFs including metal ion-dependent regulators Fur, Mur, Zur, and Nur which are responsible for homeostasis of the metal ions in the organism. For each studied TF we used relevant gene expression assays and knockout fitness dataset when available. We also applied our approach to a novel genome *D. alaskensis* to predict the binding behavior of one of its hypothetical regulators *Dde0289*. In fact, we discovered a rare type of binding motif which is structurally-weak and unexposed to most sequence-based motif finding tools. We further validated this prediction by applying the same approach to the estimated motif’s main presumed regulator (Fis) that has been annotated in *E. coli* [13]. The details of the applications and results are described as follows.

## Results and discussion

### Predictions in the model organism E. coli K12 validates our approach’s sensitivity

To assess our approach’s performance we reconstructed the regulons of LexA, PurR and Fur transcription factors which are among the well-studied regulons in *Escherichia coli K12 strain*. In literature, these transcription factor motifs are known to have palindromic sequence symmetry. The informative sites in the LexA motif are conserved in the middle of each half-site sequence, while for PurR and Fur they are mostly scattered across the motif and appear more informative near the motif half-site. In Figs. 3, 4 and 5 these structures can be seen in the estimated motifs too which are obtained by the proposed approach.

**Fig. 2:**
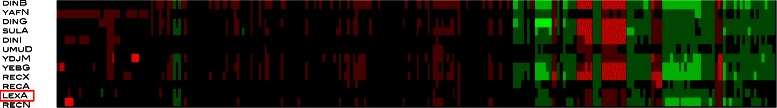
Gene expression heatmap of the estimated high-confidence set: dinB, yafN, dinG, sulA, dinI, umuD, ydjM, yebG, recX, recA, lexA, recN

We estimated a set of genes putatively co-regulated with the LexA’s coding gene *lexA*, by using the proposed biclustering method with the gene expression data from [14] corresponding to 266 experiments and the fitness data from [15] corresponding to growth rates under antibiotic stress conditions, *tetracycline, doxycycline*, and *minocycline*. The estimated high-confidence set for LexA consist of 12 putatively co-regulated genes 11 of which are members of the RegPrecise regulon. Figure 2 shows the corresponding gene expression heatmap of the estimated bicluster consisting of 12 genes and 260 conditions.

**Fig. 3:**
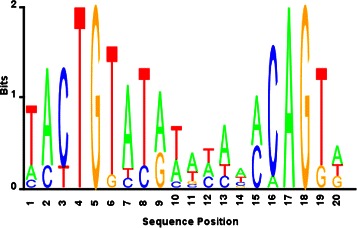
LexA motif estimated by the proposed approach

By using 300-bp upstream sequences, the motif discovery with proposed BAMBI2b algorithm yielded a 20-bp motif (Fig. 3) with the consensus sequence identical to that of the RegPrecise motif. Table 1 shows the similarity of both motifs (BAMBI2b vs RegPrecise) with the curated motif database (SwissRegulon [16]). It is seen that both motifs exhibit high similarity with the known weight matrix LexA_20-6, and the BAMBI2b estimate’s similarity is statistically more significant. By scanning *E. coli* genome with this motif we predicted 60 different putative binding sites, where 10 of them are located in the intragenic regions (open reading frames). After downstream analyses –assisted with *MicrobesOnline* prediction data for adjacent genes [17]– we identified a set of 90 genes as the putative regulon (see Additional file 2).

**Table 1:** The best hits of the estimated (BAMBI2b) vs true (RegPrecise) LexA motifs in SwissRegulon database

	BAMBI2b	RegPrecise
	motif	motif
Target ID	LexA_20-6	LexA_20-6
Optimal offset	2	2
p-value	2.05557e-11	2.97639e-09
e-value	1.99391e-09	2.88709e-07
q-value	3.96654e-09	5.74338e-07
Overlap	18	18
Query	TACTGTATATATAAACAGTA	TACTGTATATATATACAGTA
consensus		
Target	TATACTGTATATAAAAACAG	TATACTGTATATAAAAACAG
consensus		
Orientation	+	+

Comparison with RegPrecise database showed that 27 genes are the members of known LexA regulon (corresponding to 93 %), which are predicted through the same binding sites. We call this group as true positives (TP). Four novel binding sites are also found for the true positives *dinB*, *ydjM*, *ruvA*, and *lexA* (Table 2), in addition to their RegPrecise binding sites. The remaining 63 genes are not identified in RegPrecise, 17 of which have intragenic binding sites.

**Table 2:** Novel binding sites for LexA regulon

Locus	Gene	Position	Score	TFBS sequence
b0231	dinB	-111	7.6	AGCTGGATAAGCAGCAGGTG
b1728	ydjM	-52	10	CACTGTATAAAAATCCTATA
b1861	ruvA	-51	8.6	TGCTGTATGATAAAAAAATG
b4042	lexA	-88	8.7	AACTGCACAATAAACCAGAG

We analyzed this putative regulon of 90 genes for biological significance by using *Database for Annotation, Visualization and Integrated Discovery (DAVID)* [18, 19] and identified certain significant genes that are novel to RegPrecise database. A cluster of 29 genes including *alkB*, *dinJ*, *ada*, *yagL*, and *rmuC*, was annotated with the highest functional enrichment score 25.5 (see Additional file 2). Notice that *alkB*, *ada* and *yagL* were predicted via intragenic TFBSs that are excluded in RegPrecise regulon. Protein interactions (SP-PIR) indicated high significance for the group including *aklB* and *ada* for *DNA repair* and *DNA damage* terms. For the groups including *aklB*, *ada*, and *dinJ*, Gene Ontology (GO) terms *cellular response to stress*, *DNA repair*, and *response to DNA damage stimulus* were the reported enrichment terms. Table 3 shows the corresponding false discovery rates (FDRs) (Benjamini). On the other hand, *dinJ*, *yafQ*, *rpod*, *molR*, and *insK* are identified in the experimental database RegulonDB [20] as the genes regulated by LexA.

**Table 3:** Functional enrichment in reconstructed LexA regulon

Terms	aklB, ada	aklB, ada, dinJ
DNA repair (SP-PIR)	5.1E-28	
DNA damage (SP-PIR)	6.2E-28	
Cellular response to stress (GO)		7.5E-23
DNA repair (GO)		1.6E-21
Response to DNA damage stimulus (GO)		1.6E-21

**Fig. 4:**
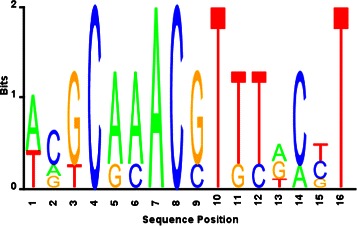
PurR motif estimated by the proposed approach

We obtained a similar performance for the putative PurR regulon. Based on the gene expression data in [21], the proposed biclustering method estimated a high-confidence set that consists of 5 true positive genes, i.e., *purR, cvpA, purC, purM,* and *purN*. From the upstream sequences, BAMBI2b discovered a 16-bp motif (Fig. 4) which is comparable to the RegPrecise motif (Table 4).

**Table 4:** The best hits of the estimated (BAMBI2b) vs true (RegPrecise) PurR motifs in SwissRegulon database

	BAMBI2b motif	RegPrecise motif
Target ID	PurR_17-3	PurR_17-3
Optimal offset	0	0
p-value	4.59804e-10	3.53226e-12
e-value	4.4601e-08	3.4263e-10
q-value	8.82639e-08	6.74503e-10
Overlap	16	16
Query consensus	ACGCAAACGTTTACCT	ACGCAAACGTTTGCGT
Target consensus	ACGCAAACGTTTTCCTT	ACGCAAACGTTTTCCTT
Orientation	+	+

After TFBS prediction and subsequent regulon reconstruction, we obtained a putative regulon consisting of 158 genes regulated via 93 non-intragenic sites (Additional file 3). Thirty-three genes are identified in the RegPrecise regulon which are regulated by the same binding sites. Our approach found 3 additional binding sites for the true positive genes, *serA, yieG(purP)*, and *yjcD* (Table 5).

**Table 5:** Novel binding sites for PurR regulon

Locus	Gene	Position	Score	TFBS sequence
b2913	serA	-95	8.1	ATATGAACGTTTGCGT
b3714	yieG	-115	8.4	ACGGCAACGATTGCGT
b4064	yjcD	-76	7.6	AAGATAACGTTTCGCT

GO annotationss for this putative regulon suggested a cluster of genes with significant functional enrichment. Among the genes, *nudB, cadA, cadB, cysZ, gltF, gltS, rhtC*, and *ygeW* were members of this cluster which are annotated by the GO term *nitrogen compound biosynthetic process* (see Additional file 3).

**Fig. 5:**
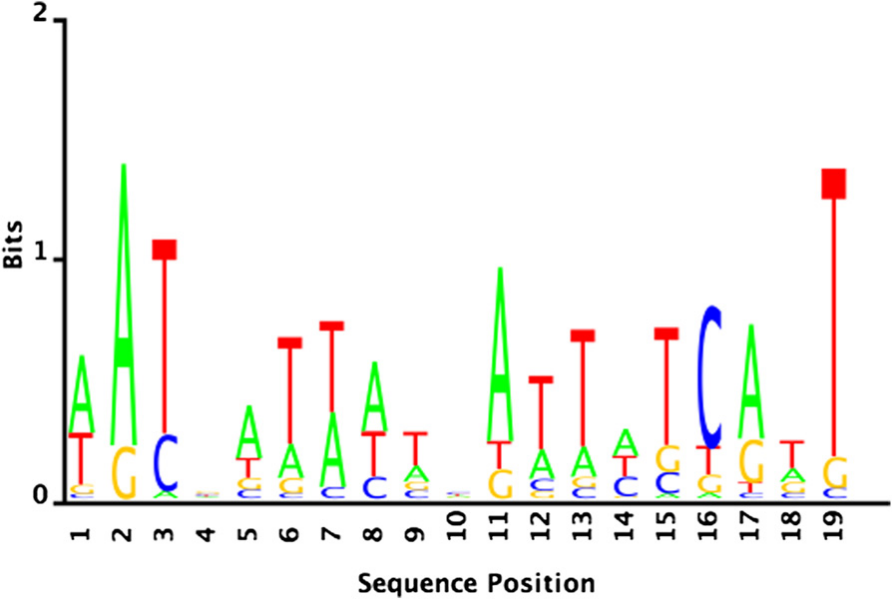
Fur motif estimated by the proposed approach

The reconstruction of Fur regulon also indicated novel predictions. We employed the gene expression data in [22], and the fitness data [15] with growth rates under antibiotic stress conditions. The proposed biclustering approach estimated a high-confidence set containing 56 genes of which 19 are members of the Fur regulon in RegPrecise database. From the upstream sequences of these 56 genes BAMBI2b discovered a 19-bp motif (Fig. 5, Table 6). Although it slightly differs from the RegPrecise’s Fur motif (i.e., the binding sites align with a 3-bp shift and have the same consensus sequence), the motif still conforms the same palindromic symmetry and is able to recover the same RegPrecise binding sites (see Additional file 4).

**Table 6:** The best hits of the estimated (BAMBI2b) vs true (RegPrecise) Fur motifs in SwissRegulon database

	BAMBI2b	RegPrecise
	motif	motif
Target ID	Fur_21-4	Fur_21-4
Optimal offset	4	1
p-value	3.01028e-07	2.00946e-10
e-value	2.91997e-05	1.94918e-08
q-value	5.77853e-05	3.00056e-08
Overlap	17	19
Query	AATGATTATCATTATCATT	GATAATGATTATCATTATC
consensus		
Target	TGATAATGATAATAATTATCA	TGATAATGATTATCATTATCA
consensus		
Orientation	-	+

After screening the *E. coli* genome, we obtained a putative regulon consisting of 236 genes that covers all identified Fur operons in the RegPrecise database. Among them 32 genes have the intragenic binding sites. The annotation analysis identified a group of 71 genes with the highest enrichment score (10.6) and associated them to the metal ion/iron related functional terms, in particular, the protein interaction terms *iron, iron transport, transport*, and *ion transport* (see Additional file 4). Among this group we pinpointed two genes *fes* and *fhuE* (Table 7) that are novel to RegPrecise database. In literature, *fes* and *fhuE* are notable for making iron available for metabolic use [23] and regulating ferrum uptake (Fe3+) via coprogen [24], respectively. Both are identified in RegulonDB as the members of Fur regulon.

**Table 7:** Functional enrichment in reconstructed Fur regulon

Terms	fes, fhuE
Iron (SP-PIR)	1.5E-16
Iron transport (SP-PIR)	2.3E-16
Transport (SP-PIR)	6.8E-15
Ion transport (SP-PIR)	1.9E-13

**Fig. 6:**
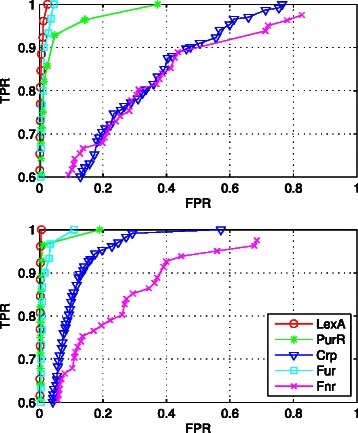
ROC of reconstruction results based on BAMBI2b-estimated motifs (above) vs RegPrecise motifs (below)

To evaluate the reconstruction accuracy in terms of specificity/sensitivity performance we implemented our approach in different datasets, i.e., LexA, PurR, Fur, Crp, and Fnr, and assessed the impact of our estimated motifs (BAMBI2b) vs true (RegPrecise) motifs in the reconstruction results. Figure 6 shows the corresponding ROC curves, where each data point represents a putative regulon with corresponding true positive (*TPR*) and false positive rate (*FPR*) in respect to the (true) regulon in RegPrecise database, i.e., $TPR = \frac {TP}{TP+FN}$, $FPR = \frac {FP}{FP+TN}$. These measures depend on the predefined site score threshold, i.e., the recovery rate (TPR) increases from left to right as the site score threshold is lowered and more true binding sites are recovered.

Since the performance of our approach depends on the complexity of the TF’s regulatory network, we expect better performance for relatively smaller regulons. It can be seen from Fig. 6 that the recovery rates are less significant for the larger regulons Crp and Fnr. This is in accord with our expectations since the Crp and Fnr family TFs are among the 7 global regulators that control 50 % of all regulated genes in *E. coli* [25].

### Results for the hypothetical proteins indicates good predictions for non-generic TF binding motifs

We used our approach to predict the motifs of hypothetical proteins of the novel organism *Desulfovibrio alaskensis* [26]. It is an anaerobic sulfate-reducing bacteria that is notable for its ability to produce hydrogen sulfide, a chemically reactive product toxic to plants, animals and humans. *Dde0289* is one of the hypothetical DNA-binding proteins in D. alaskensis, which is annotated as a Sigma-54-dependent transcriptional activator. It is presumed to belong to Fis-type helix-turn-helix motifs in literature.

**Fig. 7:**
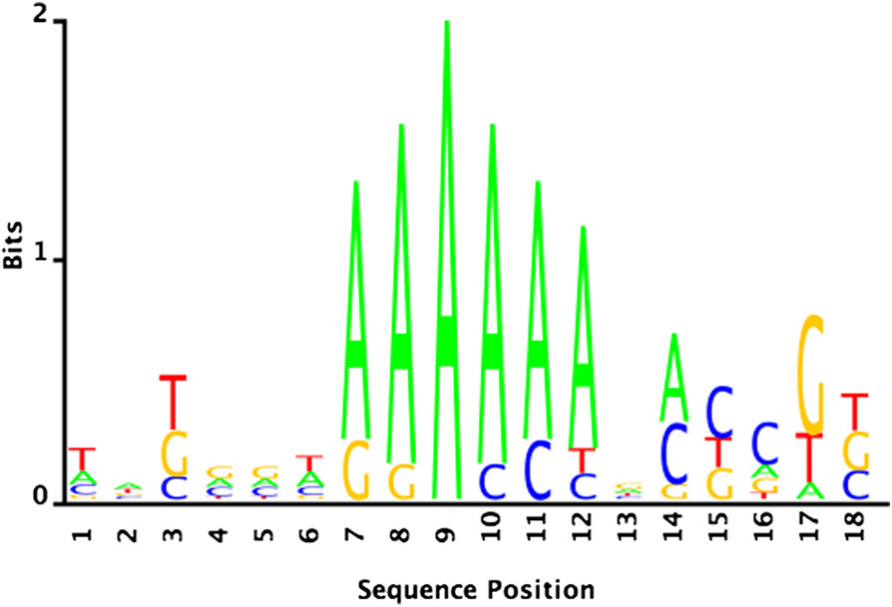
Hypothetical *Dde0289* motif estimated by the proposed approach

For this regulator, we used all available gene expressions and fitness data sets from MicrobesOnline database [27] in the proposed biclustering method. The estimated high-confidence set consisted of 11 co-expressed genes, i.e., *Dde0289, Dde0312, Dde2767, Dde2741, Dde2343, Dde1987, Dde1148, Dde2317, Dde0481, Dde2075*, and *Dde1935*. From the upstream sequences, BAMBI2b discovered the instances containing A-tracts, significantly appearing in the middle of the motif (Fig. 7).

Although such structure is not generic in TF motifs, it is known that the intrinsic sequence-dependent protein-DNA conformations can result in high-affinity binding events. A recent study proposes that A-tracts are the preferred Fis-binding sites in *E. coli*, and in particular, A_6_-tracts provide the strongest binding signals [13]. A_6_-tracts are known to induce intrinsic curvature to segments of DNA [28], whereby it enhances the local region’s exposure to transcription machinery.

**Fig. 8:**
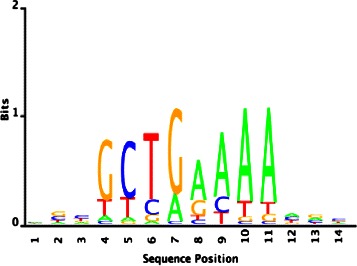
Fis motif estimated by the proposed approach

To validate our approach’s sensitivity for recovering such rare binding motifs, we reconstructed *E. coli*’s Fis motif, and compared our results with those that are deduced by the ChIP-chip binding data in [13]. We used the gene expression data set in [29], and estimated a high-confidence set of 100 genes by using the proposed biclustering method and Algorithm 1 (see Additional file 1). BAMBI2b found several motif estimates that consist of variably conserved A-tracts flanked by the G residues. In particular, the estimates partially recovered the Fis motif’s consensus sequence GCTGAAAAAA, with the highest information content conserved at GCTGAAAA (Fig. 8) which corresponds to the consensus half-site sequence of the Fis motif.

This is in accord with the findings in [13], where the different Fis motif subtypes (non-palindromic and palindromic) share the most common bases in their consensus half-site. In fact, motif comparison of our estimate with the SwissRegulon database resulted in a significant hit to one of the Fis weight matrices, i.e., Fis_26–32 (Table 8). In contrast, we used the true Fis weight matrix obtained from RegulonDB, and the motif comparison assigned this motif to the same weight matrix Fis_260–32 with a similar score.

**Table 8:** The best hits of the estimated (BAMBI2b) vs true (RegulonDB) Fis motifs in SwissRegulon database

	BAMBI2b motif	RegulonDB motif
Target ID	Fis_26-32	Fis_26-32
Optimal offset	7	3
p-value	6.29038e-06	3.21289e-08
e-value	0.000610167	3.1165e-06
q-value	0.00121382	6.16615e-06
Overlap	10	15
Query consensus	CGCTGAAAAA	GCTTATTTTTTAAGC
Target consensus	GTTCTGTTGCTGAAAAAATAACCAAA	TTTGGCTATTTTTTCAGCAACAGAAC
Orientation	+	-

### Refining the methods

We applied certain constraints to refine our predictions. Knockout fitness data sets are integrated to refine high-confidence gene set estimation by giving more biological relevance. Integrating appropriately-selected growth conditions often showed a positive effect for eliminating false positive genes. In motif discovery, we imposed the two-block motif structure to account for palindromic or inverted/direct-repeat symmetry patterns of the TFBSs. We also used a heuristic to define an optimal data feeding order to motif discovery problem based on the co-expression of local genes.

Further constraints can be imposed in the reconstruction program to limit the extent of predictions, such as searching genes only in the downstream direction by the strand which TF putatively binds. On the other hand, some restrictions could be relaxed to refine the results in particular cases. For instance, one can obtain different estimates by performing multiple runs of BAMBI2b with scrambled data order, in particular, when the co-expression patterns are not very determinative. If a reference motif is available, e.g., when reconstructing/expanding known regulons, this allows one to directly use it as the prior PWM (***θ***) in the proposed motif discovery algorithm BAMBI2b (see Additional file 1 for more detail.) Such procedures will likely reduce the negative effect of the false positive sequences which can diminish the high-confidence set.

## Conclusions

In this paper, we proposed a computational method to predict TF regulons of a single organism without relying on phylogenetic footprinting techniques. The proposed approach requires gene expression (and knockout fitness) experiments for the organism of interest, and thereby can be suitable for predicting novel TF regulons. In particular, we aimed at bacterial transcription factors by using a two-block motif model to represent the binding sites and minimizing an information-theoretic dissimilarity measure between the TFBS cores. The presented results for LexA, PurR and Fur TFs in the model organism *E. coli* showed high recovery rates for their experimentally-verified regulons. Possible extensions as additional TFBSs for the known regulon genes and new putatively regulated genes showing high biological significance are noted. Experiments with a novel organism *D. alaskensis* also showed intuitive predictions for the hypothetical regulators. In particular, we observed that our approach is sensitive enough to discover rare TF binding events by recovering structurally low-probability motifs. In the light of results reported, we conclude that a motif-based regulon inference approach can discover the organism-specific regulatory interactions on a single organism, which may be missed by current comparative genomics techniques due to their limitations.

## Methods

### Co-regulated gene set estimation

For a given TF, we first estimate a group of putatively co-regulated genes in which we seek coherent expression patterns with the given TF’s coding gene. The gene expression data sets basically serve as the problem input. This computational problem has been addressed by numerous clustering algorithms [30, 31]. Recently, biclustering methods have gained more attention for their superiority in representing co-regulation in high-dimensional data sets by grouping the genes simultaneously with appropriate set of conditions. Since the generic clustering algorithms classify genes into different functional groups by considering all data points (conditions) at once they often fail to capture true interactions, if the genes exhibit similar behavior under only some but not all conditions.

In this work, we use a biclustering algorithm to select an optimal group among the annotated genes by simultaneously choosing a subset of experiments that best captures the group’s co-expression. The optimization algorithm looks for linear coherency of the data points (i.e., genes’ expressions) with the given TF’s coding gene. This model assumes linear dependency of expression between the co-regulated gene pairs. Although such simplifications may not reflect the real underlying relationships, they often yield effective results by capturing the zero-th and first order interactions [32]. When the coherency in high-dimensional expression data becomes indiscriminate we employ the genome wide “*knockout fitness*” data as further biological evidence. The latter monitors the organism-level responses (fitness, survival rate) by exposing knockout/knockdown mutant strain libraries of genes to various experimental stress conditions [33], whereby providing the biclustering algorithm a systems-level insight.

#### Filtering out uninformative genes

After a high-confidence gene set is found we supply their upstream sequences for motif discovery. It is known that the adjacent genes are often co-regulated in local complexes (i.e., operons) and their expressions are controlled through only a few sites, hence the occurrence of binding sites in the upstream of such genes could be very sparse. So, the great majority of genes in an estimated high-confidence set may in fact belong to a few operons depending on the TF. In such cases, the upstream sequences not containing a cognate binding site will likely deteriorate the discovery of the true motif. We used a correlation-based filtering algorithm to detect those sequences that more likely contain a regulatory site of the underlying motif. Given a set of genes, by comparing each genes’ expression coherency with the TF’s coding gene the algorithm iteratively selects those that strictly follow its adjacent (preceding) gene’s co-expression pattern. (The details –i.e., Algorithm 1– are given in Additional file 1).

### Motif discovery

For the motif finding problem, we employ a Bayesian algorithm (BAMBI) [34] for discovering motifs of an unknown length and unknown number of instances in a given set of sequences. Estimating such unknown quantities as the number, length, and locations of the motif instances in each sequence is cast as a probabilistic inference problem through the use of hidden Markov model (HMM) framework. A computationally efficient sequential Monte Carlo algorithm is employed with a sampling procedure for constructing the posterior distributions of the hidden variables [35].

We modified this algorithm to capture particularly the TF motifs, i.e., by exploiting the intrinsic sequence properties such as base conservation and spatial similarity observed in the transcription factor binding sites. We called the newly proposed algorithm “BAMBI2b”. Since in most TF binding sites the bases variably contribute to the affinity of TF-DNA binding complex, defining a suitable model tailored for TFs is crucial for motif discovery [36]. We employ a “*two-block*” motif model [37] to represent the TFBS’s conserved (core) segments by a pair of “*blocks*” where the information content is mostly concentrated. The length and location of such segments within the motif are not known a priori and they are estimated within the Bayesian framework. On the other hand, most TF binding sites are known to have certain sequence similarities where the half sites (mostly cores) occur to be (i) Watson-Crick complements (palindromic symmetry), (ii) identical sequences (direct-repeat symmetry), or (iii) reversed sequences (inverted-repeat symmetry). We use an information-theoretic measure in order to estimate the correct symmetry type from the TFBS cores. The algorithm looks to find such motif instances that will minimize the sequence dissimilarity between the PWM’s corresponding blocks, whereby maximizing the intrinsic symmetry conformation. (We represent the core dissimilarity as an averaged cross-entropy distance between the base probabilities of the motif blocks – see Additional file 1 for more detail).

### Regulon reconstruction

Once the motif is established, we scan the entire genome by it for TFBS prediction. For each query sequence a binding score is calculated by a statistical significance metric using the motif’s PWM and the background nucleotide distribution. For this, we used the site recognition method presented in [7] which evaluates a possible binding site by two metrics, i.e., a likelihood-ratio (raw) score that quantifies the degree of motif’s preference in the respective site, and p-value that indicates the probability of obtaining this score (or a greater score) merely by chance. After setting a sufficient P-value threshold (0.001) and defining an intuitive log-likelihood ratio score threshold (e.g. such that the algorithm will recover the majority of the known TFBSs) we eliminate the structurally weak binding sites in our putative TFBS list, and check the remaining sites for a nearby gene appearance. Binding sequences that are located in the upstream of a gene’s 5^′^ start site are paired with those genes, and they together constitute the putative operons of the TF.

We allow bi-directional search to identify target genes on both the forward and complementary strands. For example, if a TFBS is predicted to bind on the positive strand, we look for target genes via (*i*) the site’s 5’–3’ direction on the positive strand and (*ii*) the complementary site’s 5’–3’ direction on the negative strand. Each time a gene is found, the program checks –as an option– if the adjacent genes are predicted to be in the same operon by using the operon prediction database [17], and if so the program includes them in the putative regulon.

TFBSs falling within the intragenic regions are often ignored in comparative genomics approaches due to ortholog-dependent reconstruction. Here, we allow the algorithm to look for such binding sites within the coding regions or open reading frames. As a result, this significantly improves the recovery of experimentally-verified binding sites and increases novel predictions.

### Motif comparison

We used Tomtom [38] to quantify the similarity between TF motifs. It calculates statistical measures between the given query motifs and a database of known motifs. In this study, we used the SwissRegulon’s motif database [16] for *E. coli* TFs. For each motif, we displayed the results for the best hit obtained by Tomtom in its default settings.

## Availability of supporting data

The data sets supporting the results of this article are included within the article (and its additional files).

## Additional files

Additional file 1 **Supplementary Material**. Mathematical details of the proposed methods are provided in this file. (PDF 343 kb)

Additional file 2 **Supplementary Data I**. Supplementary data for the predicted LexA regulon. (XLS 167 kb)

Additional file 3 **Supplementary Data II**. Supplementary data for the predicted PurR regulon. (XLS 233 kb)

Additional file 4 **Supplementary Data III**. Supplementary data for the predicted Fur regulon. (XLS 237 kb)

Additional file 5 **BAMBI2b software**. Source codes (C++) of the proposed motif discovery algorithm BAMBI2b are given in this package. (ZIP 122 kb)

## References

[1] Chua G, Morris Q, Sopko R, Robinson M, Ryan O, Chan E (2006). Identifying transcription factor functions and targets by phenotypic activation. Proc Natl Acad Sci USA.

[2] Gelfand M, Novichkov P, Novichkov E, Mironov A (2000). Comparative analysis of regulatory patterns in bacterial genomes. Brief Bioinf.

[3] Price M, Dehal P, Arkin A. Horizontal gene transfer and the evolution of transcriptional regulation in Escherichia Coli. Genome Biol. 2008;9. doi:10.1186/gb-2008-9-1-r4. 10.1186/gb-2008-9-1-r4 PMC2395238 18179685

[4] Kazakov A, Rodionov D, Price M, Arkin A, Dubchak I, Novichkov P (2013). Transcription factor family-based reconstruction of singleton regulons and study of the CRP/FNR, ArsR, and GntR families in Desulfovibrionales genomes. J Bacteriol.

[5] Novichkov P, Laikova O, Novichkova E, Gelfand M, Arkin A (2010). Regprecise: a database of curated genomic inferences of transcriptional regulatory interactions in prokaryotes. Nucleic Acids Res.

[6] Ashburner M, Ball C, Blake J, Botstein D, Butler H, Cherry J (2000). Gene ontology: tool for the unification of biology. Nat Genet.

[7] Frith M, Fu Y, Yu L, Chen J, Hansen U, Weng Z (2004). Detection of functional DNA motifs via statistical over-representation. Nucleic Acids Res.

[8] Dhaeseleer P (2006). How does DNA sequence motif discovery work?. Nat Biotechnol.

[9] Yang L, Zhou T, Dror I, Mathelier A, Wasserman WW, Gordan R (2014). Tfbsshape: a motif database for dna shape features of transcription factor binding sites. Nucleic Acids Res.

[10] Zhou T, Shen N, Yang L, Abe N, Horton J, Mann RS (2015). Quantitative modeling of transcription factor binding specificities using DNA shape. Proc Nat Acad Sci USA.

[11] Liu L, Jin G, Zhou X (2015). Modeling the relationship of epigenetic modifications to transcription factor binding. Nucleic Acids Res.

[12] Novichkov PS, Rodionov DA, Stavrovskaya ED, Novichkova ES, Kazakov AE, Gelfand MS (2010). Regpredict: an integrated system for regulon inference in prokaryotes by comparative genomics approach. Nucleic Acids Res.

[13] Cho B, Knight E, Barrett C, Palsson B (2008). Genome-wide analysis of Fis binding in Escherichia Coli indicates a causative role for A-/AT-tracts. Genome Res.

[14] Faith J, Hayete B, Thaden J, Mogno I, Wierzbowski J, Cottarel G (2007). Large-scale mapping and validation of Escherichia Coli transcriptional regulation from a compendium of expression profiles. PLoS Biol.

[15] Nichols RJ, Sen S, Choo YJ, Beltrao P, Zietek M, Chaba R (2011). Phenotypic landscape of a bacterial cell. Cell.

[16] Pachkov M, Erb I, Molina N, van Nimwegen E (2007). Swissregulon: a database of genome-wide annotations of regulatory sites. Nucleic Acids Res.

[17] Price M, Huang K, Alm E, Arkin A (2005). A novel method for accurate operon predictions in all sequenced prokaryotes. Nucleic Acids Res.

[18] Dennis G, Sherman B, Hosack D, Yang J, Gao W, Lane H (2003). David: database for annotation, visualization, and integrated discovery. Genome Biol.

[19] Huang D, Sherman B, Lempicki R (2009). Bioinformatics enrichment tools: paths toward the comprehensive functional analysis of large gene lists. Nucleic Acids Res.

[20] Salgado H, Peralta-Gil M, Gama-Castro S, Santos-Zavaleta A, MuÃśiz-Rascado L, GarcÃ-a-Sotelo JS (2013). RegulonDB v8.0: omics data sets, evolutionary conservation, regulatory phrases, cross-validated gold standards and more. Nucleic Acids Res.

[21] Jozefczuk S, Klie S, Catchpole G, Szymanski J, Cuadros I, Steinhauser D (2010). Metabolomic and transcriptomic stress response of Escherichia Coli. Mol Syst Biol.

[22] Masse E, Vanderpool C, Gottesman S (2005). Effect of RyhB small RNA on global iron use in Escherichia Coli. J Bacteriol.

[23] Pettis G, Brickman T, McIntosh M (1988). Transcriptional mapping and nucleotide sequence of the Escherichia Coli fepA-fes enterobactin region. Identification of a unique iron-regulated bidirectional promoter. J Biol Chem.

[24] Sauer M, Hantke K, Braun V (1990). Sequence of the fhue outer membrane receptor gene of escherichia coli k12 and properties of mutants. Mol Microbiol.

[25] Martinez A, Collado V (2003). Identifying global regulators in transcriptional regulatory networks in bacteria. Curr Opin Microbiol.

[26] Hauser LJ1, Land ML, Brown SD, Larimer F, Keller KL, Rapp-Giles BJ (2011). Complete genome sequence and updated annotation of Desulfovibrio Alaskensis G20. J Bacteriol.

[27] Dehal PS, Joachimiak MP, Price MN, Bates JT, Baumohl JK, Chivian D et al. Microbesonline: an integrated portal for comparative and functional genomics. Nucleic Acids Res. 2009. doi:10.1093/nar/gkp919. 10.1093/nar/gkp919 PMC2808868 19906701

[28] Koo H, Wu H, Crothers D (1986). DNA bending at Adenine ·Thymine tracts. Nature.

[29] Bradley M, Beach M, Koning A, Pratt T, Osuna R (2007). Effects of Fis on Escherichia Coli gene expression during different growth stages. Microbiology.

[30] Basett D, Eisen M, Boguski M (1999). Gene expression informatics– its all in your mine. Nat Genet.

[31] Bekiranov S, Gaasterland (2000). Making the most of microarray data. Nat Genet.

[32] Gan X, Liew A, Yan H (2008). Discovering biclusters in gene expression data based on high-dimensional linear geometries. BMC Bioinf.

[33] Oh J, Fung E, Price M, Dehal P, Ronald W, Giaever G (2010). A universal TagModule collection for parallel genetic analysis of microorganisms. Nucleic Acids Res.

[34] Jajamovich G, Wang X, Arkin A, Samoilov M (2011). Bayesian multiple-instance motif discovery with BAMBI: inference of recombinase and transcription factor binding sites. Nucleic Acids Res.

[35] Dong B, Wang X, Doucet A (2003). A new class of soft MIMO demodulation algorithms. Signal Process IEEE Trans.

[36] Stormo G (2000). DNA binding sites: representation and discovery. Bioinformatics.

[37] Liang K, Wang X, Anastassiou D (2008). A sequential Monte Carlo method for motif discovery. Signal Process IEEE Trans.

[38] Gupta S, Stamatoyannopoulos JA, Bailey TL, Noble WS (2007). Quantifying similarity between motifs. Genome Biol.

