# Supplementary material for: Reconstruction of novel transcription factor regulons through inference of their binding sites
Source: BMC Bioinformatics. 2015 Sep 21;16:299. doi: 10.1186/s12859-015-0685-y (PMC4576408; doi:10.1186/s12859-015-0685-y)
Supplement: Supplementary file 1 — Supplementary Material. Mathematical details of the proposed methods are provided in this file. (PDF 343 kb) [file 12859_2015_685_MOESM1_ESM.pdf]

METHODOLOGY

Reconstruction of Novel Transcription Factor Regulons Through Inference of Their Binding Sites
Supplementary Material

Abdulkadir Elmas[1], Xiaodong Wang[1*] and Michael S Samoilov[2]

Email: Abdulkadir Elmas – elmas@ee.columbia.edu; Xiaodong Wang – wangx@ee.columbia.edu; Michael S Samoilov – mssamoilov@lbl.gov

*Correspondence: wangx@ee.columbia.edu
[1]Department of Electrical Engineering, Columbia University, 500W 120th Street, 10027 New York, USA
Full list of author information is available at the end of the article
[2]Department of Bioengineering, QB3 California Institute for Quantitative Biosciences UC Berkeley, USA.

1 System model

1.1 High-confidence co-regulated gene set estimation via sparse biclustering

Given the genome-wide expression profiles we want to estimate a group of genes that are putatively co-regulated with the coding gene of a TF of interest. Co-regulation of a pair of genes can be quantified by the expression coherency across the data points (conditions).

Let $\boldsymbol{M} \in \mathcal{R}^{n \times p}$ be the gene expression data consisting of n genes and p conditions (features). Let $\boldsymbol{m}_i$ denote the i-th row of $\boldsymbol{M}$. For a given subset of features ($\subseteq \{1, ..., p\}$) , the observation pair $(\boldsymbol{m}_i, \boldsymbol{m}_j)$ can be represented as a beam around the linear regression line with slope a_{ij} and intercept b_{ij} (e.g., Fig. 1). Then the feature selection problem is to select a subset of features that minimizes the sum of residuals from the best regression.

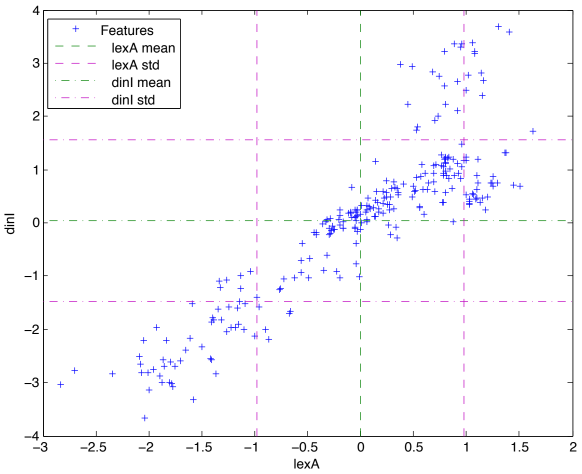

Figure 1 Scatter plot of the features corresponding to expression rates of genes lexA and dinI. The data is obtained from [1].

For the bicluster generated for the i-th observation (e.g., a TF's coding gene), feature selection can be represented by a vector, $\boldsymbol{s}_i \in \{0,1\}^p$, such that $s_{ik} = 1$ if the feature k is selected, and 0 otherwise. To avoid trivial solutions –not selecting any feature– a sparse penalty term is introduced to the problem, i.e., $\beta_1\|\mathbf{1} - \boldsymbol{s}_i\|_1$, where β_1 is the regularizer coefficient and $\mathbf{1}$ is the vector of 1s. Therefore, the feature selection problem for the i-th gene can be represented as,

$$\min_{\boldsymbol{s}_i, a_{ij}, b_{ij}} \sum_{k=1}^{p} s_{ik}(m_{ik} - a_{ij}m_{jk} - b_{ij})^2 + \beta_1\|\mathbf{1} - \boldsymbol{s}_i\|_1 \ , \tag{1}$$

where $s_{ik} \in \{0,1\}$.

To find coherent observations under the conditions given by $\boldsymbol{s}_i$ a gene selection vector $\boldsymbol{w}_i \in \{0,1\}^n$ is introduced, such that $w_{ij} = 1$ if there is a strong linear coherence between the observation pair $(\boldsymbol{m}_i, \boldsymbol{m}_j)$, and $w_{ij} = 0$ otherwise. Similarly, another sparse regularizer is added to penalize trivial biclusters, i.e., $\beta_2\|\mathbf{1} - \boldsymbol{w}_i\|_1$. To favor co-up-regulated and co-down-regulated observation samples, an importance matrix $\boldsymbol{D} \in \mathcal{R}^{n\times n\times p}$ is introduced, where $d_{ijk} \in [0,1]$ indicates the importance of the k-th feature for the observation pair $(\boldsymbol{m}_i, \boldsymbol{m}_j)$ which is computed as a function of the euclidean distance of the k-th feature to the central point $(\overline{\boldsymbol{m}}_i, \overline{\boldsymbol{m}}_j)$ in the scatter plot. Relaxing $w_{ij} \in \{0,1\}$ to $w_{ij} \in [0,1]$ and $s_{ik} \in \{0,1\}$ to $s_{ik} \in [0,1]$, the gene expression biclustering problem for the i-th gene can then be cast as,

$$\min_{\boldsymbol{w}_i, \boldsymbol{s}_i, a_{ij}, b_{ij}} \sum_j w_{ij} \sum_k s_{ik} \frac{1}{d_{ijk}} (m_{ik} - a_{ij}m_{jk} - b_{ij})^2 + \\ \beta_1\|\mathbf{1} - \boldsymbol{s}_i\|_1 + \beta_2\|\mathbf{1} - \boldsymbol{w}_i\|_1 \ , \tag{2}$$

where $w_{ij} \in [0,1]$, $s_{ik} \in [0,1]$ [2].

By optimizing (2) for a given regulatory gene i and expression matrix $\boldsymbol{M}$, one can obtain a bicluster where the genes within the bicluster exhibit high linear-coherency with each other. One may expect that the high coherency in gene expression could indicate a common transcriptional activity. However, this correlation may occur either by chance (e.g. resulting from experimental noise) or because of indirect effects underlying the experiments, thereby integration of additional biological evidence into the problem can be crucial. In particular, we can employ the fitness data to check whether the knockout strains of the bicluster genes can show similar responses (fitness) to a particular stress, i.e., we may assume that the co-regulated genes – when knocked out– are more likely to cause similar growth rates for the organism. Following this intuition, the algorithm looks to find the knock outs that have similar growth patterns under a subset of stress conditions.

Let $\boldsymbol{F} \in \mathcal{R}^{n\times q}$ be the fitness data consisting of n genes and q conditions. For the observation pair $(\mathbf{f}_i, \mathbf{f}_j)$ in $\boldsymbol{F}$ we determine the feature selection by

$$\min_{\hat{\boldsymbol{s}}_i, a_{ij}, b_{ij}} \sum_{\ell=1}^{q} \hat{s}_{i\ell}(f_{i\ell} - a_{ij}f_{j\ell} - b_{ij})^2 + \hat{\beta}_1\|\mathbf{1} - \hat{\boldsymbol{s}}_i\|_1 \ ,$$

where $\hat{s}_{i\ell} \in \{0,1\}$, and $\hat{\beta}_1$ control the feature selection in $\boldsymbol{F}$. We optimize the observation selection such that

$$\sum_{\ell} \frac{\hat{s}_{i\ell}}{\hat{d}_{ij\ell}}(f_{i\ell} - a_{ij}f_{j\ell} - b_{ij})^2 + \hat{\beta}_1\|\mathbf{1} - \hat{\boldsymbol{s}}_i\|_1 + \hat{\beta}_2\|\mathbf{1} - \boldsymbol{w}_i\|_1 \ ,$$

where $\hat{\beta}_2$ controls the gene selection and $\hat{d}_{ij\ell}$ is the importance of the ℓ-th feature for the observation $(\mathbf{f}_i, \mathbf{f}_j)$.

For a given regulatory gene i, gene expression matrix $\boldsymbol{M}$, and fitness data matrix $\boldsymbol{F}$, the biclustering problem can then be written as

$$\min_{\boldsymbol{w}_i, \boldsymbol{s}_i, \hat{\boldsymbol{s}}_i, a_{ij}, b_{ij}} \sum_j w_{ij} \Bigg(\sum_k \frac{s_{ik}}{d_{ijk}}(m_{ik} - a_{ij}m_{jk} - b_{ij})^2 + \sum_{\ell} \frac{\hat{s}_{i\ell}}{\hat{d}_{ij\ell}}(f_{i\ell} - a_{ij}f_{j\ell} - b_{ij})^2 + \beta_1\|\mathbf{1} - \boldsymbol{s}_i\|_1 + \hat{\beta}_1\|\mathbf{1} - \hat{\boldsymbol{s}}_i\|_1 + (\beta_2 + \hat{\beta}_2)\|\mathbf{1} - \boldsymbol{w}_i\|_1 \Bigg) \ , \tag{3}$$

where $w_{ij} \in [0,1]$, $s_{ik} \in [0,1]$, and $\hat{s}_{i\ell} \in [0,1]$. Since (3) is not jointly convex in $\boldsymbol{W}, \boldsymbol{S}, \hat{\boldsymbol{S}}, \boldsymbol{A}$, and $\boldsymbol{B}$, we propose an iterative procedure for solving it as follows.

Initialization: $\boldsymbol{A}$ is initialized to $\mathbf{1} \cdot \mathbf{1}^T$ since the genes' expression levels (rows) are normalized to [0,1]. $\boldsymbol{B}$ is normalized as the linear regression line for each observation $(\boldsymbol{m}_i, \boldsymbol{m}_j)$ passing through the central point $(\overline{\boldsymbol{m}}_i, \overline{\boldsymbol{m}}_j)$ with slope a_{ij}. $\boldsymbol{S}$ and $\hat{\boldsymbol{S}}$ are initialized as selecting the feature points whose importance distance to regression line remain within a certain threshold, i.e., $s_{ik} = 1$ if the k-th data point's distance to the central point $(\overline{\boldsymbol{m}}_i, \overline{\boldsymbol{m}}_j)$ is larger than some predefined threshold. $\boldsymbol{W}$ is computed from initial values of $\boldsymbol{S}, \hat{\boldsymbol{S}}, \boldsymbol{A}$, and $\boldsymbol{B}$. Each variable is then updated through optimizing its cost function when the other variables are fixed.

Updating $\boldsymbol{W}$: When $\boldsymbol{S}, \hat{\boldsymbol{S}}, \boldsymbol{A}$, and $\boldsymbol{B}$ are fixed (3) becomes a convex linear objective of $\boldsymbol{W}$, i.e.,

$$\min_{w_{ij}} \ w_{ij} \Bigg(\sum_{k=1}^{p} \frac{s_{ik}}{d_{ijk}}(m_{ik} - a_{ij}m_{jk} - b_{ij})^2 + \sum_{\ell=1}^{q} \frac{\hat{s}_{i\ell}}{\hat{d}_{ij\ell}}(f_{i\ell} - a_{ij}f_{j\ell} - b_{ij})^2 - (\beta_2 + \hat{\beta}_2) \Bigg) \quad s.t. \quad w_{ij} \in [0,1], \tag{4}$$

which can be solved as $w_{ij} = 1$ if $\left(\sum_{k=1}^{p} \frac{s_{ik}}{d_{ijk}}(m_{ik} - a_{ij}m_{jk} - b_{ij})^2 + \sum_{\ell=1}^{q} \frac{\hat{s}_{i\ell}}{\hat{d}_{ij\ell}}(f_{i\ell} - a_{ij}f_{j\ell} - b_{ij})^2\right) < (\beta_2 + \hat{\beta}_2)$, and $w_{ij} = 0$ otherwise.

Updating $\boldsymbol{S}$: When $\boldsymbol{W}, \hat{\boldsymbol{S}}, \boldsymbol{A}$, and $\boldsymbol{B}$ are fixed, the objective function is a convex (linear) function of S, i.e.,

$$\min_{s_{ik}} s_{ik} \left(\sum_j \frac{w_{ij}}{d_{ijk}}(m_{ik} - a_{ij}m_{jk} - b_{ij})^2 - \beta_1 \right) \quad s.t. \quad s_{ik} \in [0,1], \tag{5}$$

then $\boldsymbol{S}$ can be updated in closed form such that $s_{ik} = 1$ if $\sum_j \frac{w_{ij}}{d_{ijk}}(m_{ik} - a_{ij}m_{jk} - b_{ij})^2 < \beta_1$, and $s_{ik} = 0$ otherwise.

Updating $\hat{\boldsymbol{S}}$*:* Similarly, when $\boldsymbol{W}, \boldsymbol{S}, \boldsymbol{A}$, and $\boldsymbol{B}$ are fixed, the objective function is convex in $\hat{\boldsymbol{S}}$, i.e.,

$$\min_{\hat{s}_{i\ell}} \hat{s}_{i\ell}\left(\sum_j \frac{w_{ij}}{\hat{d}_{ij\ell}}(f_{i\ell} - a_{ij}f_{j\ell} - b_{ij})^2 - \hat{\beta}_1\right) \quad s.t. \quad \hat{s}_{i\ell} \in [0,1], \tag{6}$$

and $\hat{S}$ is updated in closed from such that $\hat{s}_{i\ell} = 1$ if $\sum_j \frac{w_{ij}}{\hat{d}_{ij\ell}}(f_{i\ell} - a_{ij}f_{j\ell} - b_{ij})^2 < \hat{\beta}_1$, and $\hat{s}_{i\ell} = 0$ otherwise.

Updating $\boldsymbol{A}$ *and* $\boldsymbol{B}$*:* When $\boldsymbol{W}, \boldsymbol{S}$, and $\hat{\boldsymbol{S}}$ are fixed, $\boldsymbol{A}$ and $\boldsymbol{B}$ can be solved as a least-squares linear regression problem.

The procedure iterates by updating $\boldsymbol{W}, \boldsymbol{S}, \hat{\boldsymbol{S}}, \boldsymbol{A}$, and $\boldsymbol{B}$ until the objective function converges. After convergence, we check if the bicluster have sufficient members (intuitively between 5-50 genes) to discover a regulatory motif from their upstream sequences. Before motif discovery, a pre-processing may be necessary for refining those inputs. As one can expect, the obtained high-confidence set may contain false positive genes and the corresponding upstream sequences can thereby deteriorate the motif estimation. Even some true positive genes may lack the corresponding regulatory sites in their upstream sequences which makes the motif finding problem ill-defined. Next, we will demonstrate a filtering procedure to eliminate those potentially uninformative genes from the obtained set.

1.2 Filtering high-confidence gene set

We aim to eliminate genes that presumably lack the regulatory patterns in their upstream, which is generally observed in gene groups that belong to the same operon. If the reference knowledge about the TF is available (e.g., consensus sequence, motif, etc.) we could easily detect such genes by scanning their upstream regions. However, for novel genomes such prior information is usually not available. On the other hand, for most sequenced genomes the operon predictions data are available. In such data, the adjacently co-regulated genes are grouped into operons whereby the common regulatory sites are located in the upstream of the first-in-operon genes. Using this information, an intuitive elimination procedure could be first including only the first-in-operon genes and then sequentially filtering in the downstream genes as necessary. Our filtering procedure is outlined as follows.

Assume that the high-confidence set $\mathcal{H}$ consist of the genes appearing in three different operons named as a, b, and c, e.g., $\mathcal{H} = \{a_1, a_2, a_3, b_1, b_2, c_1, c_2, c_3\}$, where a_1 is the first-in-operon gene in the operon a and a_2 is the second-in-operon gene, and so forth for the other operons b and c.

- Determine the first-in-operon genes $\mathcal{F} = \{a_1, b_1, c_1\}$ and the downstream genes $\mathcal{D} = \{a_2, a_3, b_2, c_2, c_3\}$ in the high-confidence set by using the operon predictions database [3]. Start the new high-confidence set with only the first-in-operon genes, e.g., $\mathcal{H} = \mathcal{F} = \{a_1, b_1, c_1\}$.
- For each operon in $\mathcal{D}$, calculate the difference S in co-expression score between a pair of adjacent genes, where the co-expression score is defined as *"the*

coherence of gene expressions (correlation) with the given TF gene", e.g., $S(a_2) = score(a_1) - score(a_2)$, where $score(x) = Corr(x, TFgene)$.

- Add the downstream genes with score $S < threshold$, and check if $(\boldsymbol{i})$ there are ≥ 10 genes in $\mathcal{H}$ or $(\boldsymbol{ii})$ 50% of the downstream genes are filtered in.
- If $(\boldsymbol{i})$ or $(\boldsymbol{ii})$ satisfies, return $\mathcal{H}$. Otherwise, continue adding more downstream genes by incrementing the threshold within a given range then comparing the next set of gene pairs in the downstream (see *Algorithm 1*).

Algorithm 1 Downstream gene filter

Require: set $\mathcal{H} = \mathcal{F}$, and $\mathcal{D}_0 = \mathcal{D}$
Require: set $i = 2$, and $threshold = min(thresholdRange)$
1: **for** every operon $x \in \mathcal{D}$, e.g., $x \in \{a, b, c\}$, **do**
2: Calculate $S(x_i) = score(x_{i-1}) - score(x_i)$
3: **if** $S(x_i) < threshold$ **then**
4: $\mathcal{H} = \mathcal{H} \cup x_i$
5: $\mathcal{D} = \mathcal{D} \setminus x_i$
6: **end if**
7: **end for**
8: **if** $|\mathcal{H}| \geq 10$ **then**
9: return $\mathcal{H}$
10: **else if** $|\mathcal{D}| \leq \frac{|\mathcal{D}_0|}{2}$ **then**
11: return $\mathcal{H}$
12: **else**
13: $threshold = threshold + \frac{0.1}{1+|H_{new}|-|H_{old}|}$
14: **if** $threshold >= max(thresholdRange)$ **then**
15: $i = i + 1$
16: $threshold = min(thresholdRange)$
17: **end if**
18: jump to 1
19: **end if**

Setting a sensible *thresholdRange* is important, e.g., starting with a negative value may be intuitive. A negative S occurs when the gene's correlation score is higher than that of a preceding gene, which indicates that the gene is likely directly-regulated by a TF and the regulatory pattern could be present in the corresponding gene's upstream region.

1.3 Motif discovery with BAMBI2b

Genes regulated by the same TF usually share a common nucleotide pattern in their promoter regions where the TF specifically recognizes this pattern and binds. The locations of such TF binding sites and the properties of the corresponding motif can be computationally derived by motif discovery algorithms. Here, we employ the Bayesian Algorithm for Multiple Biological Instances of motif discovery –BAMBI [4]– to estimate the unknown motif for the putatively co-regulated genes obtained in the previous section.

BAMBI is designed to discover nucleotide motifs via estimating the number, length and locations of the binding instances in a collection of sequences, where each sequence may contain one, several or no instances of the motif. The algorithm is based on the sequential Monte Carlo (SMC) technique which processes one input sequence at a time efficiently in a sequential manner. The system is modeled as an HMM, where the hidden state at time t corresponds to the current input's state vector $\boldsymbol{x}_t$ composed by the number of motif instances n_t and their locations in the current sequence (observation) $\boldsymbol{s}_t$. As the iteration proceeds, given t sequences $\boldsymbol{S}_t = \{\boldsymbol{s}_1, \ldots, \boldsymbol{s}_t\}$, BAMBI aims to identify the number of motif instances and their

locations for each sequence, i.e., $\boldsymbol{X}_t = \{\boldsymbol{x}_1, \ldots, \boldsymbol{x}_t\}$. Since the distribution of the state vectors given observations $p(\boldsymbol{X}_t|\boldsymbol{S}_t)$ is not known and the samples are usually not available for this posterior, an approximation is implemented by taking weighted samples (particles), $\boldsymbol{X}_t^k$, k=1, ..., K, from a trial density $q(\boldsymbol{X}_t|\boldsymbol{S}_t)$ within the sequential importance sampling (SIS) framework [5]. The sequential algorithm is obtained by setting $q(\boldsymbol{X}_t^k|\boldsymbol{S}_t) = q(\boldsymbol{X}_{t-1}^k|\boldsymbol{S}_{t-1})q(\boldsymbol{x}_t^k|\boldsymbol{X}_{t-1}^k, \boldsymbol{S}_t)$, and computing the sample weights

$$w_t^k \propto w_{t-1}^k \frac{p(\boldsymbol{s}_t|\boldsymbol{X}_t^k, \boldsymbol{S}_{t-1})p(\boldsymbol{x}_t^k|\boldsymbol{X}_{t-1}^k, \boldsymbol{S}_{t-1})}{q(\boldsymbol{x}_t^k|\boldsymbol{X}_{t-1}^k, \boldsymbol{S}_t)}. \tag{7}$$

A resampling procedure [6] is employed to reduce the increasing variance of ineffective samples with very small weights resulting from the phenomenon known as the degeneracy problem [7].

The length-M motif is described as a $4\times M$ PWM, i.e., $\boldsymbol{\theta} = [\boldsymbol{\theta}_1, \ldots, \boldsymbol{\theta}_M]$, where $\boldsymbol{\theta}_j$, $j = 1, \ldots, M$, represents the unknown nucleotide distributions (i.e., A, C, G, T/U) in each position of the motif that will be estimated. Since each promoter sequence can have one, several or no binding site (instance) of the motif their abundance also needs to be estimated. Assuming N instances per sequence as the upper bound, the distribution of the number of instances is described by an unknown vector $\boldsymbol{\lambda} = [\lambda_0 \ldots \lambda_N]$, where λ_j is the proportion of sequences with j instances of the motif.

TF binding sites have intrinsic properties that should be taken into account in the model, such as the location of conserved bases in the half-sites and their symmetry. We modified BAMBI to incorporate such properties of the TFBS. In particular, we can use the general two-block model [8] to describe the palindromic or direct/inverted repeat patterns in the half-sites. Estimating the location and size of the core segments will be introduced later under the "*Two-block model*" section. To model the motif symmetry we consider three types, i.e., (i) palindromic, (ii) inverted-repeat and (iii) direct-repeat symmetry. We represent the distribution of the symmetry of instances as an unknown vector, i.e., $\boldsymbol{\sigma} = [\sigma_0, \sigma_1, \sigma_2]$, where σ_0 represents the proportion of instances with palindromic symmetry, and σ_1 (σ_2) represents the proportion of instances with inverted(direct)-repeat symmetry.

At each step t, given the first t sequences $\boldsymbol{S}_t$ the sequential algorithm estimates the number of instances in each sequence and their locations, $\boldsymbol{X}_t$, within the Bayesian framework. The motif matrix $\boldsymbol{\theta}$ is modeled as *Dirichlet* random vectors. The distribution of the number of instances $\boldsymbol{\lambda}$ and the distribution of the symmetry of instances $\boldsymbol{\sigma}$ are also modeled as *Dirichlet* random vectors. A sufficient statistic $\boldsymbol{\alpha}^t$ is used to characterize the distribution of $\boldsymbol{\theta}$, and it can be updated sequentially, i.e., $\boldsymbol{\alpha}^t = \{\alpha_{m,j}^t\}_{m=1\ldots M}^{j=1\ldots 4}$, where $\boldsymbol{\alpha}_m^t = [\alpha_{m,1}^t \ldots \alpha_{m,4}^t]^T$ is the parameters for the m-th *Dirichlet* distribution corresponding to the m-th column in PWM $\boldsymbol{\theta}_m$. Then the parameters of $p(\boldsymbol{\theta}|\boldsymbol{X}_t, \boldsymbol{S}_t)$ can be sequentially updated from $p(\boldsymbol{\theta}|\boldsymbol{X}_{t-1}, \boldsymbol{S}_{t-1})$ as

$$\alpha_{m,j}^t = \alpha_{m,j}^{t-1} + n(\boldsymbol{a}_{t,x_t}(m)),$$

where $\boldsymbol{a}_{t,x_t}$ is the subsequence of M letters in the sequence $\boldsymbol{s}_t$ whose location is given by $\boldsymbol{x}_t$. Similarly, a sufficient statistic is selected for the distribution of the number

of instances $\boldsymbol{\lambda}$ which is *Dirichlet* distributed with parameters $\boldsymbol{\gamma}^t = [\gamma_0^t \dots \gamma_N^t]$. The sequential update is obtained by $\boldsymbol{\gamma}^t = \boldsymbol{\gamma}^{t-1} + \boldsymbol{j}(\boldsymbol{x}_t)$, where $\boldsymbol{j}(\boldsymbol{x}_t)$ is a vector of zeros except for a 1 indicating the number of instances of the motif in the t-th sequence. A sufficient statistic for the distribution of the symmetry of instances $\boldsymbol{\sigma}$ is also defined. $\boldsymbol{\sigma}$ is a *Dirichlet* distribution with parameters $\boldsymbol{\delta}^t = [\delta_0^t \ \delta_1^t \ \delta_2^t]$ which can be updated sequentially by $\boldsymbol{\delta}^t = \boldsymbol{\delta}^{t-1} + u(\boldsymbol{a}_{t,x_t})$, where

$$u(\boldsymbol{a}_{t,x_t}) = [u_0 \ u_1 \ u_2] = [\sum_{m=1}^{M/2} I_{\{\boldsymbol{a}_{t,x_t}(m) = \overline{\boldsymbol{a}_{t,x_t}(M-m)}\}}, \sum_{m=1}^{M/2} I_{\{\boldsymbol{a}_{t,x_t}(m) = \boldsymbol{a}_{t,x_t}(M-m)\}}, \sum_{m=1}^{M/2} I_{\{\boldsymbol{a}_{t,x_t}(m) = \boldsymbol{a}_{t,x_t}(m+\frac{M}{2})\}}].$$

$I_{\{.\}}$ is the indicator function which takes the value 1 if a nucleotide pair observed in $\boldsymbol{a}_{t,x_t}$ obeys the given symmetry type, palindromic, inverted-repeat, or direct-repeat, and the operator $\overline{()}$ takes the Watson-Crick complement of the given letter.

The influence of these variables can be averaged out in the SIS procedure, whereby the measurement model depends on $\boldsymbol{\theta}$ and $\boldsymbol{\sigma}$, and the state transition depends on $\boldsymbol{\lambda}$. The importance distribution can be chosen with $q(\boldsymbol{x}_t|\boldsymbol{X}_{t-1}^k, \boldsymbol{S}_t) = p(\boldsymbol{x}_t|\boldsymbol{X}_{t-1}^k, \boldsymbol{S}_t)$, where

$$\begin{aligned} p(\boldsymbol{x}_t|\boldsymbol{X}_{t-1}^k, \boldsymbol{S}_t) &\propto p(\boldsymbol{s}_t|\boldsymbol{x}_t, \boldsymbol{X}_{t-1}^k, \boldsymbol{S}_{t-1}) p(\boldsymbol{x}_t|\boldsymbol{X}_{t-1}^k, \boldsymbol{S}_{t-1}) \\ &= \int p(\boldsymbol{s}_t|\boldsymbol{x}_t, \boldsymbol{X}_{t-1}^k, \boldsymbol{S}_{t-1}, \boldsymbol{\theta}) p(\boldsymbol{\theta}|\boldsymbol{x}_t, \boldsymbol{X}_{t-1}^k, \boldsymbol{S}_{t-1}) d\boldsymbol{\theta} \\ &\quad \int p(\boldsymbol{x}_t|\boldsymbol{X}_{t-1}^k, \boldsymbol{S}_{t-1}, \boldsymbol{\lambda}) p(\boldsymbol{\lambda}|\boldsymbol{X}_{t-1}^k, \boldsymbol{S}_{t-1}) d\boldsymbol{\lambda}). \end{aligned} \tag{8}$$

Then, for each particle, k=$1, \dots, K$, the importance weight can be updated as

$$w_t^k \propto w_{t-1}^k \sum_{\boldsymbol{x}_t} p(\boldsymbol{s}_t|\boldsymbol{x}_t, \boldsymbol{X}_{t-1}^k, \boldsymbol{S}_{t-1}) p(\boldsymbol{x}_t|\boldsymbol{X}_{t-1}^k, \boldsymbol{S}_{t-1}). \tag{9}$$

Given the PWM $\boldsymbol{\theta}$, the background distribution $\boldsymbol{\theta}_0$, the state $\boldsymbol{x}_t$ at time t, and the symmetry distribution $\boldsymbol{\sigma}$, we can express in closed-form the likelihood of the sequence $\boldsymbol{s}_t$ in (8) as

$$\begin{aligned} &\int p(\boldsymbol{s}_t|\boldsymbol{x}_t, \boldsymbol{X}_{t-1}^k, \boldsymbol{S}_{t-1}, \boldsymbol{\theta}) p(\boldsymbol{\theta}|\boldsymbol{x}_t, \boldsymbol{X}_{t-1}^k, \boldsymbol{S}_{t-1}) d\boldsymbol{\theta} \\ &\qquad = \boldsymbol{\theta}_0^{\boldsymbol{n}(\boldsymbol{a}_{t,x_t}^c)} \prod_{m=1}^{M} \mathbb{E}[\boldsymbol{\theta}_m^{\boldsymbol{n}(\boldsymbol{a}_{t,x_t}(m))} p(\boldsymbol{a}_{t,x_t}|\boldsymbol{\theta})], \end{aligned} \tag{10}$$

where $\boldsymbol{\theta}^{\boldsymbol{n}}$=$\prod_{j=1}^4 \theta_j^{n_j}$, $\boldsymbol{n}(\boldsymbol{a})$=$[n_1, \dots, n_4]$ with n_j, j=$1, \dots, 4$, being the number of times the j-th letter appears in the instance $\boldsymbol{a}$, and $\boldsymbol{a}_{t,x_t}^c$ is the sequence in $\boldsymbol{s}_t$ resulting from removing the instance $\boldsymbol{a}_{t,x_t}$.

The last term in (10) is the likelihood of the instance $\boldsymbol{a}_{t,x_t}$ given the PWM $\boldsymbol{\theta}$, and the motif's underlying symmetry $\boldsymbol{\sigma}$. The influence of the symmetry can be averaged out by computing the integral $p(\boldsymbol{a}_{t,x_t}|\boldsymbol{\theta}) = \int p(\boldsymbol{a}_{t,x_t}|\boldsymbol{\theta},\boldsymbol{\sigma})p(\boldsymbol{\sigma}|\boldsymbol{\theta})d\boldsymbol{\sigma}$. For a given instance $a_{t,i}$, as a measure of dissimilarity (asymmetry) we use the Kullback-Leibler (KL) divergence between the PWM's base variations indicated by the symmetry type observed in $a_{t,i}$. Since the KL divergence is a non-symmetric distance measure we define a symmetrized similarity metric and write the closed-from expression for $p(\boldsymbol{a}_{t,x_t}|\boldsymbol{\theta})$ as follows.

$$\begin{aligned} p(\boldsymbol{a}_{t,x_t}|\boldsymbol{\theta}) &= \int p(\boldsymbol{a}_{t,x_t}|\boldsymbol{\theta},\boldsymbol{\sigma})p(\boldsymbol{\sigma}|\boldsymbol{\theta})d\boldsymbol{\sigma} = p(\boldsymbol{a}_{t,x_t},\boldsymbol{\theta},s)\mathbb{E}[\sigma_s] \\ &= \frac{1}{1+H(\boldsymbol{\theta}^s_a,\boldsymbol{\theta}^s_{\bar{a}})+H(\boldsymbol{\theta}^s_{\bar{a}},\boldsymbol{\theta}^s_a)}\,\frac{\delta_s}{\sum_{s=0}^{2}\delta_s}, \end{aligned} \tag{11}$$

where

$$\begin{aligned} H(\boldsymbol{\theta}^0_a,\boldsymbol{\theta}^0_{\bar{a}}) &= \sum_{m=1}^{M/2}\sum_{j=1}^{4} -I_{\{\boldsymbol{a}_{t,x_t}(m)=\overline{\boldsymbol{a}_{t,x_t}(M-m)}\}} \\ &\qquad\qquad \theta_m(j)\log(\theta_{M-m}(4-j)), \\ H(\boldsymbol{\theta}^1_a,\boldsymbol{\theta}^1_{\bar{a}}) &= \sum_{m=1}^{M/2}\sum_{j=1}^{4} -I_{\{\boldsymbol{a}_{t,x_t}(m)=\boldsymbol{a}_{t,x_t}(M-m)\}} \\ &\qquad\qquad \theta_m(j)\log(\theta_{M-m}(j)), \\ H(\boldsymbol{\theta}^2_a,\boldsymbol{\theta}^2_{\bar{a}}) &= \sum_{m=1}^{M/2}\sum_{j=1}^{4} -I_{\{\boldsymbol{a}_{t,x_t}(m)=\boldsymbol{a}_{t,x_t}(m+\frac{M}{2})\}} \\ &\qquad\qquad \theta_m(j)\log(\theta_{m+\frac{M}{2}}(j)), \end{aligned}$$

and $s{=}0,1,2$ refers to the given symmetry type, i.e., palindromic, inverted-repeat, and direct-repeat, respectively. We normalize these cross entropy measures by the number of symmetric bases in $\boldsymbol{a}_{t,x_t}$, i.e., $u_s(\boldsymbol{a}_{t,x_t})$. By restricting the calculation to only block sites $\mathcal{B}$, i.e., $m \in \mathcal{B} \subset \{1,\ldots,M/2\}$, the density is then multiplied by $\frac{u_s(\boldsymbol{a}_{t,x_t})}{1+|\mathcal{B}|}$ and log-normalized to average out the influence of the block length $|\mathcal{B}|$, which replaces (11) by

$$\begin{aligned} &p(\boldsymbol{a}_{t,x_t}|\boldsymbol{\theta}) \\ &\quad = \log(\tau + \frac{\frac{u_s(\boldsymbol{a}_{t,x_t})^2}{1+|\mathcal{B}|}}{1+H(\boldsymbol{\theta}^s_a,\boldsymbol{\theta}^s_{\bar{a}})+H(\boldsymbol{\theta}^s_{\bar{a}},\boldsymbol{\theta}^s_a)})\frac{\delta_s}{\sum_{s=0}^{2}\delta_s}, \end{aligned} \tag{12}$$

where τ is a constant to avoid taking $\log(0)$ and serves as a tolerance threshold for the asymmetric instances, i.e., the larger is τ the algorithm is more likely to sample an (asymmetric) instance that possess a larger cross-entropy. By using (12) the expectation term in (10) can be approximated as

$$\begin{aligned} &\mathbb{E}[\boldsymbol{\theta}_m^{\boldsymbol{n}(\boldsymbol{a}_{t,x_t}(m))}p(\boldsymbol{a}_{t,x_t}|\boldsymbol{\theta})] \\ &\approx \boldsymbol{\theta}_m^{\boldsymbol{n}(\boldsymbol{a}_{t,x_t}(m))}\log(\tau + \frac{\frac{u_s(\boldsymbol{a}_{t,x_t})^2}{1+|\mathcal{B}|}}{1+H(\boldsymbol{\theta}^s_a,\boldsymbol{\theta}^s_{\bar{a}})+H(\boldsymbol{\theta}^s_{\bar{a}},\boldsymbol{\theta}^s_a)})\frac{\delta_s}{\sum_{s=0}^{2}\delta_s}. \end{aligned} \tag{13}$$

The second integral in (8) is computed analogously.

$$\int p(\boldsymbol{x}_t|\boldsymbol{X}_{t-1}^k, \boldsymbol{S}_{t-1}, \boldsymbol{\lambda})p(\boldsymbol{\lambda}|\boldsymbol{X}_{t-1}^k, \boldsymbol{S}_{t-1})d\boldsymbol{\lambda}$$
$$= p(i_1 \dots i_n|n)\mathbb{E}[\lambda_n] = p(i_1 \dots i_n|n)\frac{\gamma_n^{t-1}}{\sum_{i=0}^{N}\gamma_i^{t-1}}, \quad (14)$$

where $p(i_1 \dots i_n|n)$ is distributed uniformly.

Given a set of promoter sequences $\boldsymbol{S}_t = \{\boldsymbol{s}_1, \dots, \boldsymbol{s}_t\}$, the BAMBI2b algorithm for discovering symmetric two-block motifs is then summarized as follows.

For each sequence, and for each particle,

- Construct the importance distribution by computing (8,10,13,14) and enumerating all possible sample extensions

  $$\boldsymbol{X}_t^k(n, i_1, \dots, i_n) = [\boldsymbol{X}_{t-1}^k[ni_1 \dots i_n]^T];$$

- Draw sample $\boldsymbol{x}_t^k$ from the importance distribution and set

  $$\boldsymbol{X}_t^k = [\boldsymbol{X}_{t-1}^k \boldsymbol{x}_t^k];$$

  and update the particle weight using (9);
- Update the sufficient statistics $\boldsymbol{\alpha}^t$, $\boldsymbol{\gamma}^t$, and $\boldsymbol{\delta}^t$; then, resample if needed.

Two-block model The bacterial TF binding sites are usually symmetric in sequence where the half-sites obey a palindromic or direct/inverted repeat symmetry. The more informative bases in each half-site are generally conserved in the middle. One can model such motif structure as a pair of informative symmetric sequences (blocks $\mathcal{B}$) separated by a run of uninformative bases (gap), and a pair of uninformative flanker sequences ($\mathcal{F}$) located at each end (Fig. 2). To estimate this structure within the SMC framework we used the class-based resampling scheme presented in [6] and augmented the state vector as $\boldsymbol{z}_t = [\boldsymbol{x}_t^T, m, b, f]^T$, where m, b, and f are the parameters for motif length M, block length B, and flanker length F, respectively. The classes are enumerated as $\Lambda_{M,B,F} = \{(M, B, F) \mid B \in \{1, \dots, \frac{M}{2}\},\ F \in \{0, \dots, \frac{M}{2} - B\}\}$, where each class represents a different scenario of the possible motif structures. The Bayesian algorithm for the two-block motif model is similar to the single-block model given in [8] except that the closed-form expressions are modified for the non-block region, i.e., background distribution $\boldsymbol{\theta}_0$ is used for the uninformative bases $\{1, \dots, M\} \setminus \mathcal{B}$.

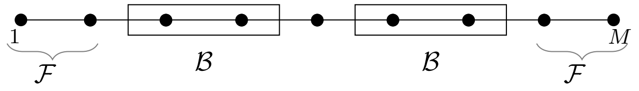

Figure 2 The two-block structure of a length-M motif described by the blocks ($\mathcal{B}$) and the flankers ($\mathcal{F}$).

The class-based resampling procedure is summarized as follows.

- Choose the number of particles for each class according to a multinomial distribution with parameters

$$P_{m,b,f} \sim \{\hat{P}(m,b,f|\boldsymbol{S}_t)\}_{(m,b,f)\in\Lambda_{M,B,F}}. \tag{15}$$

- For every class $\forall\gamma \in \Lambda_{M,B,F}$, if $P_\gamma < P_{thresh}$ then set $P_\gamma = P_{thresh}$.
- Reduce the number particles starting from the class with the largest number particles until $\sum_\gamma P_\gamma = P$.
- For every class draw P_γ new particles from the set of previous particles with probabilities proportional to their weights.
- Assign equal weights to each new sample within a class, $w_t^k = \hat{P}(m,b,f|\boldsymbol{S}_t)/P_{m,b,f}$.

Optimal data feeding

Because of the sequential nature of BAMBI2b, the order in which the sequences are utilized have a direct impact on the accuracy of estimation. An optimal data feeding should then be defined in order to prioritize the most useful sequences to supply to BAMBI2b. The downstream genes can be supplied in the same order in which they are filtered in by *Algorithm 1*.

To rank-order the first-in-operon genes $\mathcal{F}$, we can use a similar approach. The genes that are expressed more coherently with the given transcription factor may indicate a proximity to the TF's binding instances. Following this intuition, we feed the upstream sequences of the first-in-operon genes $\{x \in \mathcal{F}\}$ in a decreasing order based on the correlation scores, i.e., $score(x) = Corr(x, TFgene)$, as follows. Let $\boldsymbol{M} \in \mathcal{R}^{|\mathcal{F}|\times p}$ be the gene expression matrix corresponding to $|\mathcal{F}|$ high-confidence genes and p different conditions. Suppose that $\boldsymbol{m}_i \in \mathcal{R}^p$ is the vector of expression rates for the given transcription factor gene located in the i-th row of $\boldsymbol{M}$. The correlation scores for the genes $x = 1, \ldots, |\mathcal{F}|$, are calculated by Pearson's correlation

$$score(x) = \frac{\sum_{k=1}^{p}(m_{ik} - \overline{\boldsymbol{m}_i})(m_{xk} - \overline{\boldsymbol{m}_x})}{\sqrt{\sum_{k=1}^{p}(m_{ik} - \overline{\boldsymbol{m}_i})^2 \sum_{k=1}^{p}(m_{xk} - \overline{\boldsymbol{m}_x})^2}},$$

where $\overline{\boldsymbol{m}_i}$ and $\overline{\boldsymbol{m}_x}$ are empirical means of the expression vectors $\boldsymbol{m}_i$ and $\boldsymbol{m}_x$ of the genes i and x, respectively. The upstream sequences are then arranged in the order of descending absolute scores $|score(x)|$ of the corresponding genes in $\mathcal{F}$.

Author details

[1]Department of Electrical Engineering, Columbia University, 500W 120th Street, 10027 New York, USA.
[2]Department of Bioengineering, QB3 California Institute for Quantitative Biosciences UC Berkeley, 1700 4th St #214, 94720 California, USA.

References

1. Faith, J., Hayete, B., Thaden, J., Mogno, I., Wierzbowski, J., Cottarel, G., Kasif, S., Collins, J., Gardner, J.: Large-scale mapping and validation of Escherichia Coli transcriptional regulation from a compendium of expression profiles. PLoS Biol **5** (2007)
2. Shi, Y., Liao, X., Zhang, X., Lin, G., Schuurmans, D.: Sparse learning based linear coherent bi-clustering. Algorithms in Bioinformatics **7534**, 346–364 (2012)
3. Price, M., Huang, K., Alm, E., Arkin, A.: A novel method for accurate operon predictions in all sequenced prokaryotes. Nucleic Acids Res **33**, 880–892 (2005)
4. Jajamovich, G., Wang, X., Arkin, A., Samoilov, M.: Bayesian multiple-instance motif discovery with BAMBI: inference of recombinase and transcription factor binding sites. Nucleic Acids Res **39**, 146 (2011)
5. Dong, B., Wang, X., Doucet, A.: A new class of soft MIMO demodulation algorithms. Signal Processing, IEEE Transactions on **51**, 2752–2763 (2003)

6. Vercauteren, T., Guo, D., Wang, X.: Joint multiple target tracking and classification in collaborative sensor networks. Selected Areas in Communications, IEEE Journal on **23**, 714–723 (2005)
7. Doucet, A., Wang, X.: Monte Carlo methods for signal processing: a review in the statistical signal processing context. Signal Processing Magazine, IEEE **22**, 152–170 (2005)
8. Liang, K., Wang, X., Anastassiou, D.: A sequential Monte Carlo method for motif discovery. Signal Processing, IEEE Transactions on **56**, 4496–4507 (2008)
