# Supplementary material for: Reconstruction of novel transcription factor regulons through inference of their binding sites
Source: BMC Bioinformatics. 2015 Sep 21;16:299. doi: 10.1186/s12859-015-0685-y (PMC4576408; doi:10.1186/s12859-015-0685-y)
Supplement: Supplementary file 5 — BAMBI2b software. Source codes (C++) of the proposed motif discovery algorithm BAMBI2b are given in this package. (ZIP 122 kb) [file 12859_2015_685_MOESM5_ESM.zip › BAMBI2b software/Readme.pdf]

BAMBI-2b

Bacterial TF binding sites are usually symmetrical in sequence where the conserved (core) segments (blocks) are separated by a stretch of uninformative nucleotides and may be shifted from both ends (Figure 1). BAMBI-2b can estimate the length and location of such segments to better capture symmetric TF motifs.

Files: The file main.cpp needs to be compiled. This file has two dependencies: database.h and database.cpp, which must be in the same directory with the file main.cpp. To compile the program type "make" in command line within the same directory. The executable "BAMBI2b" will be generated.

The input sequences should be given in fasta format. The example data (sequences.fasta) includes the upstream sequences of a set of *E. coli* genes which have similar gene expression profiles in certain experiments, i.e., $dinB$, $yafN$, $dinG$, $sulA$, $dinI$, $umuD$, $ydjM$, $yebG$, $recA$, $lexA$, and $recN$, respectively.

Usage: ./BAMBI2b -i sequences.fasta -o bambi_motifs.txt -P 10000 -LM 19 -UM 21 -LB 1 -UB 5 -LF 0 -UF 3 -r0 1 -r1 300 -s0 1 -s1 1 -s2 1 -d 0.25,0.26,0.25,0.24 -n 2 -sm

- i : (REQUIRED) (Path to) Input file name (in fasta format)
- o : (optional) Output file name (default: bambi_motifs.txt)
- P : (optional) The number of samples to estimate the motif PWM from the sequence set. It is recommended to use 50 times the length of the longest input sequence (50 $\times$ (max sequence length))
- LM : (optional) Lower limit for motif length M (default: 16)
- UM : (optional) Upper limit for motif length (default: 26)
- LB : (optional) Lower limit for the length of conserved block $\mathcal{B}$ (default: 0)
- UB : (optional) Upper limit for the length of conserved block $\mathcal{B}$ (default: UM/2)
- LF : (optional) Lower limit for the length of flanking uninformative sequence (default: 0)
- UF : (optional) Upper limit for the length of flanking uninformative sequence (default: UM - 2)
- r0 : (optional) The prior information regarding the amount of instances of the motif in each sequence. Unless specific knowledge is available, it is recommended to use 1 for r0 and to set r1 to be the average length of the input sequences (default: 1)
- r1 : (optional) (default: average length of input sequences)
- s0 : (optional) Expected number of instances with palindromic symmetry per input sequence (default: 1)
- s1 : (optional) Expected number of instances with inverted-repeat symmetry per input sequence (default: 1)
- s2 : (optional) Expected number of instances with direct-repeat symmetry per input sequence (default: 1)

- d : (optional) Background nucleotide distribution (default: 0.25,0.25,0.25,0.25)
- n : (optional) Number of repeated runs (default: 1)
- sm : (optional) Flag for searching minor (secondary) sites via maximum likelihood estimation (default: false)
- op : (optional) Block similarity metric. Use the option 2 for a faster block symmetry estimation. Option 2 ignores the default cross-entropy calculations between blocks, and only counts the symmetric base-word ratios. (default: 1)

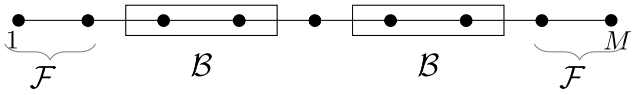

FIGURE 1. The two-block structure of a length-M motif described by the blocks ($\mathcal{B}$) and the flankers ($\mathcal{F}$).
